# Amide Proton Transfer-Weighted (APTw) Imaging of Intracranial Infection in Children: Initial Experience and Comparison with Gadolinium-Enhanced T1-Weighted Imaging

**DOI:** 10.1155/2020/6418343

**Published:** 2020-05-16

**Authors:** Hong Zhang, Xiaolu Tang, Yanqiu Lv, Di Hu, Jihang Sun, Yan Wang, Jinyuan Zhou, Yun Peng

**Affiliations:** ^1^Department of Radiology, Beijing Children's Hospital, Capital Medical University, National Center for Children's Health, Beijing, China; ^2^Division of MR Research, Department of Radiology, Johns Hopkins University, Maryland, USA

## Abstract

**Purpose:**

To evaluate the performance of amide proton transfer-weighted (APTw) imaging against the reference standard of gadolinium-enhanced T1-weighted imaging (Gd-T1w) in children with intracranial infection.

**Materials and Methods:**

Twenty-eight pediatric patients (15 males and 13 females; age range 1-163 months) with intracranial infection were recruited in this study. 2D APTw imaging and conventional MR sequences were conducted using a 3 T MRI scanner. Kappa (*κ*) statistics and the McNemar test were performed to determine whether the hyperintensity on APTw was related to the enhancement on Gd-T1w. The sensitivity, specificity, positive predictive value (PPV), and negative predictive value (NPV) of APTw imaging to predict lesion enhancement were calculated.

**Result:**

In twelve patients with brain abscesses, the enhancing rim of the abscesses on the Gd-T1w images was consistently hyperintense on the APTw images. In eight patients with viral encephalitis, three showed slight spotted gadolinium enhancement, while the APTw image also showed a slight spotted high signal. Five of these patients showed no enhancement on Gd-T1w and isointensity on the APTw image. In eleven patients with meningitis, increased APTw signal intensities were clearly visible in gadolinium-enhancing meninges. Sixty infectious lesions (71%) showed enhancement on Gd-T1w images. The sensitivity and specificity of APTw were 93.3% (56/60) and 91.7% (22/24). APTw demonstrated excellent agreement (*κ* = 0.83) with Gd-T1w, with no significant difference (*P* = 0.69) in detection of infectious lesions.

**Conclusions:**

These initial data show that APTw MRI is a noninvasive technique for the detection and characterization of intracranial infectious lesions. APTw MRI enabled similar detection of infectious lesions to Gd-T1w and may provide an injection-free means of evaluation of intracranial infection.

## 1. Introduction

Intracranial infection in children continues to be a worldwide health problem, particularly in poor and developing countries. The incidence of acute encephalitis syndrome in children is estimated to be 10.5-13.8/100,000 [1]. The case fatality rate is 30%, and one-third of survivors develop neurological disabilities [2]. The Global Burden of Disease network (WHO) estimated that meningitis caused approximately 422,900 deaths and encephalitis caused 143,500 deaths in 2010 [3]. Because of the potential for rapid deterioration and causing devastating short- and long-term neurologic sequels, timely diagnosis is of the utmost importance. Magnetic resonance imaging (MRI) is the imaging modality of choice for the diagnosis and therapeutic surveillance of pediatric intracranial infection [4–6]. Currently, widely used pulse sequences are T2-weighted, T1-weighted, fluid-attenuated inversion recovery (FLAIR), diffusion-weighted imaging (DWI), and gadolinium-enhanced T1-weighted imaging (Gd-T1w) in clinical MR imaging studies. The clinical diagnosis of intracranial infection in children mainly relies on either morphological changes or contrast-enhancement characteristics. However, recently, scientific evidence has been mounting that traces of gadolinium remain in the brain, skin, bone, liver, and other organs in patients with normal renal function [7–16]. Use of gadolinium-based contrast agents (GBCAs) is also limited in patients with renal dysfunction because of the risk of nephrogenic systemic fibrosis [17, 18]. This is particularly important for children with intracranial infection, because multiple enhanced MRI scans are usually required in these patients.

Amide proton transfer (APT) imaging [19, 20] is a chemical exchange saturation transfer (CEST) [21–23]-based molecular MRI technique, by which endogenous mobile proteins and peptides (such as those dissolved in the cytoplasm [24]) can be detected. The semisolid macromolecules in the more solid environment of the cell, such as those in the nucleus and the membrane, are detectable by conventional magnetization transfer (MT) imaging (quantified by the MT ratio or MTR) [25]. Previous studies have indicated that APT-weighted (APTw) signals could be used to assess the spatial extent and pathological grade of several human tumors (due to overexpressed mobile protein and peptide concentrations) [26–34]. Recently, one group has demonstrated that APTw contrast was significantly higher in tumors and infective mass lesions compared with normal tissue regions. This occurs due to increased cellular protein and peptide contents in lesion regions [35].

In this study, we explored intracranial infection in children using protein-based APTw imaging at 3 T. We hypothesize that APTw MRI may be a sensitive biomarker for intracranial infection (due to increased mobile, cytosolic protein, and peptide concentration). The goal of our study was to evaluate the performance of APTw imaging against the reference standard of Gd-T1w MRI in children with intracranial infection.

## 2. Materials and Methods

### 2.1. Subjects

This study was approved by the ethics committee of Beijing Children's Hospital. Written, informed consent was given by all the children's parents prior to participation to this study. From February 2014 to December 2016, 35 patients with suspected intracranial infection were enrolled. Of the 35 patients, three were excluded due to small lesion sizes (<3 mm), and four were excluded due to motion artifacts during the MRI scan. As a result, 28 patients (15 males and 13 females; age range 1-163 months; Table 1) were included in this study. For viral encephalitis, the data from 8 age- and sex-matched normal controls (5 males and 3 females; age range 15-82 months) were selected from our previous study [36] for comparison study. The infective organism was identified by blood, cerebrospinal fluid (CSF), or specific immunological serum test. Children younger than five years of age were sedated with oral 10% chloral hydrate (0.3–0.5 ml/kg) before the MRI scans.

### 2.2. MRI Protocol

All subjects were imaged on a 3-Tesla MRI system (Achieva, Philips Medical Systems), using a dual-channel body coil for transmission and an eight-channel sensitivity-encoding coil for reception. Pencil-beam, second-order shimming was used. APTw and MT imaging scans were incorporated into a standard clinical MRI examination protocol for the brain (field of view, 230 mm × 190 mm; slice thickness, 5 mm), including axial T2-weighted (repetition time, 3000 ms; echo time, 100 ms), T1-weighted and Gd-T1w (repetition time, 2308 ms; echo time, 13 ms), fluid-attenuated inversion recovery (FLAIR) and gadolinium-enhanced FLAIR (Gd-FLAIR) (repetition time, 7000 ms; echo time, 120 ms; inversion recovery time, 2200 ms), and diffusion-weighted (repetition time, 7000 ms; echo time, 120 ms; b factors, 0 and 1000 s/mm^2^). Gd-T1w was the last sequence acquired.

APTw/MT imaging was performed using a 2D single-slice sequence, based on a pseudocontinuous wave, off-resonance radiofrequency irradiation (saturation duration, 200 ms × 4; interpulse delay, 10 ms; block pulses; average power level, 2 *μ*T), and a single-shot, turbo-spin-echo readout: repetition time, 3000 ms; fast spin echo with 2 segments, 50; field of view, 230 mm × 190 mm; matrix, 144 × 95, reconstructed to be 400 × 400; slice thickness, 5 mm. We used a multioffset, multiacquisition APTw/MT imaging protocol, similar to previous studies [36–39]. 31 offsets were used: 0, ±0.25, ±0.5, ±0.75, ±1 (2), ±1.5 (2), ±2 (2), ±2.5 (2), ±3 (2), ±3.25 (2), ±3.5 (8), ±3.75 (2), ±4 (2), ±4.5, ±5, ±6, and 15.6 ppm (The values in parentheses are the number of acquisitions, which was one if not specified. 0 ppm corresponds to the water proton resonance). We acquired a separate *S*_0_ image without radiofrequency (RF) saturation for signal normalization. We had 31 offsets and 63 images. One slice through the largest apparent lesion area was acquired, and the slice coincided exactly with one of the standard MRI slice acquired (T2-weighted, T1-weighted, FLAIR, DWI, and Gd-T1w). The acquisition time was about 2 minutes 40 seconds.

### 2.3. Image Processing and Analysis

The imaging analysis was performed using the Interactive Data Language (IDL; ITT Visual Information Solutions). To reduce possible motion artifacts during the scanning, the acquired APTw and MT image series for each slice was registered to the saturated image at 3.5 ppm [40], which was first registered with the corresponding slice on the standard MRI images acquired. The registration was performed with the analysis of functional neuroimaging software (AFNI [41]; NIH/NIMH), using a rigid-body transformation of three degrees of freedom. The measured MT spectra (*S*_sat_/*S*_0_, in which *S*_sat_ and *S*_0_ are the signal intensities with and without selective radiofrequency irradiation, plotted as a function of saturation frequency offset, relative to water) were corrected for B_0_ field heterogeneity effects through their centering on a pixel-by-pixel basis, as reported before [36–39, 42]. MTR was defined according to the equation: MTR = 1‐*S*_sat_/*S*_0_. Conventional MTR images were calculated with the saturated images at 15.6 ppm (2 kHz).

CEST imaging is quantified through the magnetization transfer ratio (MTR = 1–*S*_sat_/*S*_0_) asymmetry (MTR_asym_) analysis with respect to the water resonance [39]: MTR_asym_(offset) = MTR(+offset) − MTR(−offset) = [*S*_sat_(−offset) − *S*_sat_(+offset)]/*S*_0_. Notably, at the offset of 3.5 ppm, we have MTR_asym_(3.5 ppm) = APTR + MTR_asym_′(3.5 ppm), where APTR is the proton transfer ratio for the amide protons associated with mobile cellular proteins and peptides in tissue, and MTR′_asym_ consists of various nuclear overhauser enhancement (NOE) effects of the upfield nonexchangeable protons (such as aliphatic protons) of cellular macromolecules and metabolites [43, 44], including the inherent MTR_asym_ of the solid-phase magnetization transfer effect [39]. The calculated MTR_asym_(3.5 ppm) was defined as the APTw value. The APTw image was displayed in color using a window of −4% to 4%.

Two radiologists (H.Z. and Y.P., with 7 and 15 years of experience, respectively, in pediatric brain imaging) performed a consensus interpretation of all images. The infectious lesions (including brain abscess and viral encephalitis) were first labeled on conventional MR with their sizes being no less than 3 mm for better delineation on APTw and Gd-T1w. For meningitis, two radiologists jointly reviewed the images to confirm whether abnormal sulcal or dural signal was present on conventional MR prior to intravenous contrast administration. All the lesions were determined as either enhancing or nonenhancing on Gd-T1w. On APTw, the signal intensity of the lesions was determined as either hyperintense, isointense, or hypointense to the contralateral normal-appearing brain tissue (CNABT). The foci on Gd-T1w (either enhancing or nonenhancing) were taken as the gold standard for the diagnosis, and the signal intensity (hyperintense or nonhyperintense) of each lesion on APTw was compared to the status of enhancement on Gd-T1w.

For brain abscesses, one ROI covering the whole area of the Gd-enhancing rim of the abscess on the Gd-T1w image was drawn. The median voxel number of the ROIs was 189 (range, 92–268). The perifocal edema and CNABT were also analyzed. For viral encephalitis, two ROIs in bilateral encephalitic lesions (basal ganglia, thalamus, or cerebral cortex) were drawn manually on the FLAIR images. For normal controls, two ROIs were also drawn in the same regions as patients with viral encephalitis.

### 2.4. Statistical Analysis

All data were analyzed using the statistical package SPSS for Windows (Version 17.0). The average APTw and MTR signal intensities were calculated for each patient. The results were presented as the format of the mean ± standard deviation (SD). One-way analysis of variance with post hoc tests was performed for comparing multiple values of parameters for each tissue type. Tukey's post hoc tests were used if the *P* value resulting from tests for homogeneity of variance was greater than or equal to 0.05. Otherwise, Games-Howell post hoc tests would be employed if *P* < 0.05. To assess differences in the average APTw or MTR signal intensities between viral encephalitis and normal control groups, paired Student's *t*-test was performed. APTw and MTR values between brain abscess and viral encephalitis were analyzed by an independent-sample *t*-test. Kappa (*κ*) statistics and the McNemar test were performed to determine whether the hyperintensity on APTw was related to the enhancement on Gd-T1w. For *κ*, values were defined as [45]: excellent, *κ* > 0.75; fair to good, *κ* = 0.40–0.75; and poor, *κ* < 0.40. The sensitivity, specificity, positive predictive value (PPV), and negative predictive value (NPV) of the APTw to predict lesion enhancement were calculated. Statistical significance was accepted at *P* < 0.05.

## 3. Results

### 3.1. Brain Abscess

**Figure 1:**
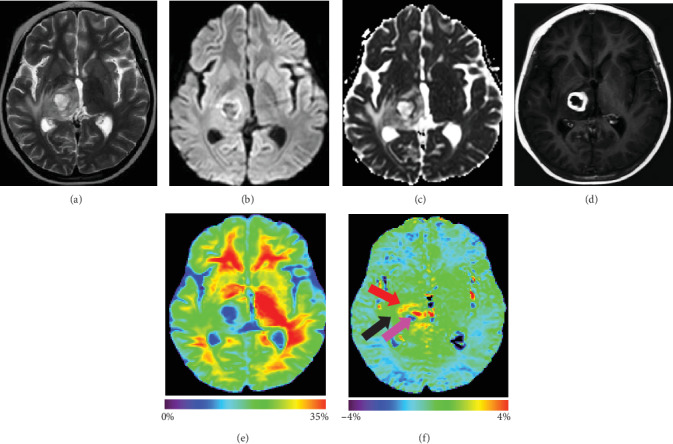
APTw, MTR, and conventional MR images for a 7-year-old girl with tuberculous abscess. T2w image (a) shows an irregular lesion with surrounding edema in the right thalamus. DWI (b) and ADC map (c) show restricted diffusion in part of the lesion. Gd-T1w image (d) demonstrates an enhancing rim with a nonenhancing necrotic center. The MTR (e) signals are low in the gadolinium-enhancing rim of the lesion and perifocal edema. APTw image (f) shows that the enhancing rim (red arrow) is hyperintense, while the necrotic region (pink arrow) and perifocal edematous area (black arrow) have low and equal APTw signals.

**Figure 2:**
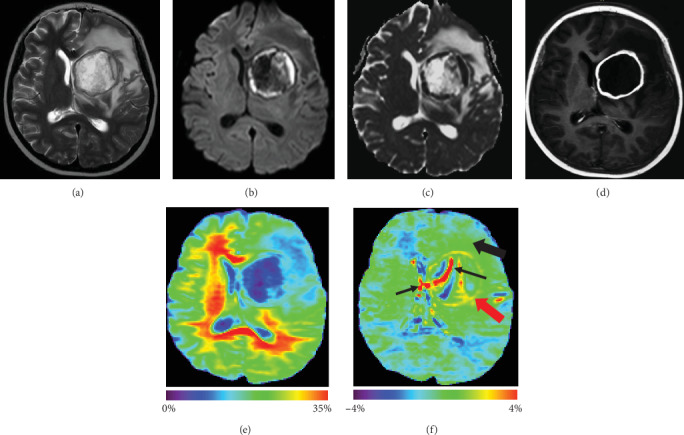
APTw, MTR, and conventional MR images for a 10-year-old girl with pyogenic abscess. T2w image (a) shows a round lesion with surrounding edema in the left basal ganglia region. DWI (b) and ADC map (c) show restricted diffusion in part of the lesion. Gd-T1w image (d) demonstrates an enhancing rim with a nonenhancing necrotic center. The MTR (e) signals are low in the gadolinium-enhancing rim of the lesion and perifocal edema. The APTw (f) signal is high in the gadolinium-enhancing rim of the lesion (red arrow), compared to the perifocal edematous area (black arrow) and CNABT. Note the alternating APTw hyper- or hypointensity in the liquefactive necrotic area and ventricles (black thin arrows).

Twelve patients suffered from brain abscesses (two with tuberculous abscesses, eight with pyogenic abscesses, and two with fungal abscesses) with a total of 24 cysts. All these brain abscesses appeared hyperintense on T2-weighted images with a peripheral hypointense rim and extensive surrounding vasogenic edema. Postcontrast T1-weighted images showed strong rim enhancement. The enhancing rim of the abscesses consistently had markedly increased APTw signal intensity (Figures 1 and 2).  Figure 1 shows APTw, MTR, and conventional MR images for a patient with tuberculous abscess. The gadolinium-enhancing rim on the postcontrast T1-weighted image was hyperintense on the APTw image, compared with perifocal edema and CNABT. The cystic areas within the abscess cavity demonstrated iso- to hypointense signal on the APTw image. The gadolinium-enhancing rim of the lesion and perifocal edema demonstrated a hypointense signal on the MTR image.  Figure 2 shows APTw, MTR, and conventional MR images for a patient with a pyogenic abscess. Similar to Figure 1, the gadolinium-enhancing rim on the postcontrast T1-weighted image showed high signal on the APTw image, compared with perifocal edema and CNABT. The gadolinium-enhancing rim of the lesion and perifocal edema demonstrated hypointense signal on the MTR image. Some parts of the cystic areas within the abscess cavity demonstrated alternating hyper- or hypointensity on the APTw image. This uncorrected artifact perhaps is caused by the local motion or large B_0_ deviation in ventricles or in liquefactive necrosis.

For all brain abscesses (*n* = 24), the average APTw image intensities were significantly higher in the gadolinium-enhancing rim of the lesion than in the perifocal edema and CNABT (2.24% versus 1.10% versus 0.49%; *P* < 0.001; Table 2), whereas the MTR intensities were significantly lower in the gadolinium-enhancing rim of the lesion than in the CNABT (15.28% versus 20.38%; *P* < 0.001; Table 2). MTR values between gadolinium-enhancing rim and perifocal edema showed no significant differences (*P* = 0.786; Table 2).

### 3.2. Viral Encephalitis

**Figure 3:**
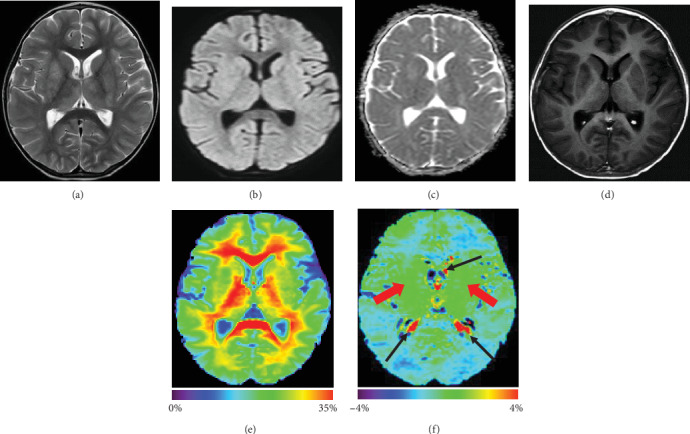
APTw, MTR, and conventional MR images for a 3-year-old boy with viral encephalitis. T2w image (a) demonstrates symmetric increased signal intensity involving bilateral basal ganglia and thalami. DWI (b) and ADC map (c) show no restricted diffusion. The lesions show no enhancement on Gd-T1w (d) and isointensity on MTR (e) and APTw (f) images (red arrows). Note the presence of artifacts in the ventricles (black thin arrows).

**Figure 4:**
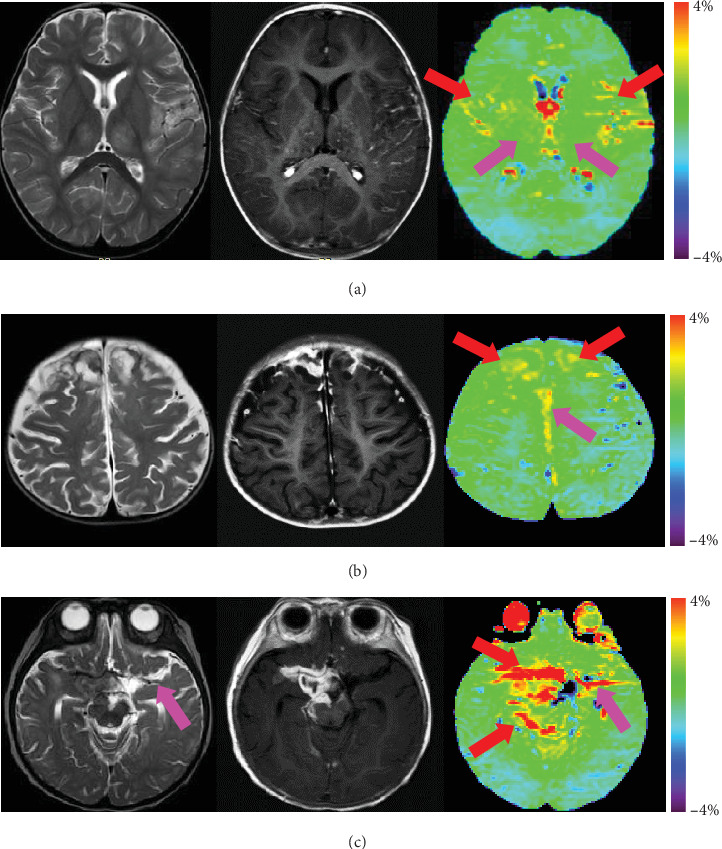
APTw and conventional MR images for three patients with meningitis. (a) T2w, Gd-T1w, and APTw images for a 17-month-old girl with viral meningoencephalitis. T2w image demonstrates symmetric increased signal intensity involving bilateral thalami and temporal lobes. The Gd-T1w image shows slight spotted enhancement in bilateral thalami and leptomeningeal enhancement in the bilateral temporal regions. The gadolinium-enhancing lesions (pink arrows) and leptomeninges (red arrows) show slight hyperintensity on the APTw image. (b) T2w, Gd-T1w, and APTw images for a 9-month-old girl with pyogenic meningitis. Gd-T1w image demonstrates leptomeningeal enhancement in the frontal regions and adjacent cerebral falx. Consistent with Gd-T1w, the APTw image shows hyperintensity in the frontal regions (red arrows) and adjacent cerebral falx (pink arrow). (c) T2w, Gd-T1w, and APTw images for a 7-month-old boy with tuberculous meningitis. Gd-T1w image shows enhancing subarachnoid space lesions in the right basilar cisterns, with extension into right sylvian vallecula, ambient cistern, and quadrigeminal cistern. The enhancing leptomeninges also show hyperintensity on the APTw image (red arrows). Note the APTw hyperintensity in the left sylvian vallecula and temporal lobe (pink arrow). T2w image verifies the presence of the left middle cerebral artery (pink arrow) in the same area.

In eight patients with viral encephalitis, there were lesions in the basal ganglia and thalami as well as gyral swelling and edema, characterized by T2-weighted/FLAIR hyperintense signal. Of these patients, three showed slight spotted gadolinium enhancement, and five did not.  Figure 3 shows APTw, MTR, and conventional MR images for a patient with viral encephalitis. T2-weighted image showed symmetric hyperintense lesions in the basal ganglia and thalami bilaterally. DWI and apparent diffusion coefficient (ADC) map show no restricted diffusion in the same areas. The lesions showed no gadolinium enhancement and APTw isointensity (compared with the normal-appearing brain tissue). Therefore, no obvious lesion can be found using either the APTw or postcontrast T1-weighted image.  Figure 4(a) shows APTw and conventional MR images for a patient with viral meningoencephalitis. The lesions showed thalami and temporal gyral swelling and edema bilaterally, characterized by T2-weighted hyperintense signal. The lesions in the thalami showed slight spotted gadolinium enhancement, while the APTw image also showed slight spotted high signal. These results mean that the isointensity signal on the APTw image is consistent with the non-Gd-enhancing area on Gd-T1w image.

All the encephalitic lesions were used for quantitative analysis. The results showed that the average APTw and MTR values were not significantly different between viral encephalitis and normal control groups (0.92% versus 0.82%; 25.98% versus 26.80%; *P* > 0.05). We further applied APTw and MT imaging to distinguish between brain abscess and viral encephalitis. The comparison showed that the average APTw intensities of the brain abscess (gadolinium-enhancing rim) were significantly higher than those of viral encephalitis (2.24% versus 0.92%; *P* < 0.001; Table 3). The average MTR intensities of the brain abscess (gadolinium-enhancing rim) were significantly lower than those of viral encephalitis (15.28% versus 25.98%; *P* < 0.001; Table 3).

### 3.3. Meningitis

Abnormal meningeal enhancement was noted in all patients with tuberculous (*n* = 4), pyogenic (*n* = 4), and viral meningitis (*n* = 3). Three patients had viral meningoencephalitis. The gadolinium-enhancing meninges on the postcontrast T1-weighted images were hyperintense on the APTw images in all patients with meningitis.  Figure 4 shows APTw and conventional MR images for three patients with meningitis. In all patients, abnormal leptomeninges were visible on the APTw images as a mild to moderate hyperintense signal, consistent with thickened pia-arachnoid, which showed enhancement after administration of contrast material. The results show that APTw MR imaging can be used to aid in detection of abnormal meningeal enhancement.

### 3.4. Agreement between APTw and Gd-T1w MR Findings

Twenty-eight pediatric patients with 84 infectious lesions (24 brain abscesses, 28 viral encephalitic lesions, and 32 meningeal lesions) were included (Table 4). APTw images demonstrated excellent agreement with Gd-T1w images (*κ* = 0.83), and the detection of infectious lesions was not significantly (*P* = 0.69) different from Gd-T1w images. APTw images provided excellent sensitivity (93.3%) and specificity (91.7%). Of 84 infectious lesions, 6 (7.1%) were discrepant between APTw and Gd-T1w images. These discrepancies were analyzed by both observers in consensus to try to explain the mismatches. Four of these discrepancies were explained by small enhancing abscesses on Gd-T1w that could not be seen on APTw. Two of these discrepancies were false positive on APTw sequence. In two meningeal lesions, APTw abnormalities were clearly visible but not on Gd-T1w images, which were considered to represent large vessels (Figure 4(c)).

## 4. Discussion

Intracranial infection in children is often life-threatening with devastating consequences. Neuroimaging plays a critical role in visualization of typical lesion patterns which allows for not only rapid diagnosis and subsequent therapeutic strategies but also monitoring of treatment response [46–48]. For the detection of intracranial infection, contrast-enhanced conventional MR imaging has been widely regarded as the most sensitive imaging study and has thus become the standard method of imaging in the clinic [5, 46, 49]. However, GBCAs have contraindications, such as increased risk for developing nephrogenic systemic fibrosis in patients with severe renal impairment and allergy to GBCAs [17, 18]. Furthermore, gadolinium retention in the brain, skin, bone, liver, and other organs has been reported [7–16]. To date, no alternative method of imaging has been shown to be an adequate substitute for gadolinium-enhanced MRI in patients with intracranial infection. APTw MRI, which uses image contrast based on CEST, could be a useful noninvasive technique for evaluation of pediatric intracranial infection, as it provides indirect measurements of mobile proteins and peptides [24]. However, till date, there are no studies available on APTw imaging of intracranial infection in children.

Our study demonstrates the feasibility of APTw MRI to reveal signals from intracranial infection that are unique compared to conventional MRI sequences. Results of the current study agree with a previous study [35], demonstrating that the average APTw signal was significantly higher in the gadolinium-enhancing rim of the brain abscess than in the perifocal edema and CNABT. This hyperintense rim on APTw MRI may be due to the inflammatory cellular infiltrate, granulomas, and gliosis [50, 51], which could cause an increase in cellularity, thus leading to an increase in APTw-detectable mobile, cytosolic protein and peptide concentration [35, 52, 53]. In addition to lesion cellular proteins, another possible contribution to the increased APTw signal in the Gd-enhancing rim of the abscess is increased vascularity, as the blood contains high concentrations of hemoglobin and albumin [20]. Consistent with previous reports [54–56], this study found that the gadolinium-enhancing rim of the brain abscess had been shown to have a lower MTR than CNABT. Conventional MT imaging (quantified by MTR) is sensitive to semisolid macromolecules in the more solid environment of the cell (such as lipids and proteins in the myelin, membrane, and nucleus). Pathological tissues usually have decreased MTR compared to normal parenchyma [55]. On the other hand, APTw imaging was designed to detect mobile proteins in biological tissues. Unlike APTw, there was no statistically significant difference in MTR between gadolinium-enhancing rim and perifocal edema. It shows that APTw is likely more sensitive to detect brain abscess than the MTR approach. For viral encephalitis, most of the lesions showed no enhancement on Gd-T1w and isointensity on APTw images. Of 28 viral encephalitic lesions, only six encephalitic lesions showed slight spotted gadolinium enhancement and slight spotted high signal on APTw image. Compared to normal brain tissue, there was no significant increase in APTw-detectable mobile, cytosolic protein and peptide concentration in encephalitic lesions. The average MTR values were also not significantly different between viral encephalitis and normal control groups. Furthermore, the present study showed that the APTw value is higher in the brain abscess and MTR value is higher in viral encephalitis. APTw and MT could be used to distinguish between brain abscess and viral encephalitis. Meningitis is an infectious/inflammatory infiltration of the leptomeninges (pia and arachnoid mater) [57]. Meningitis is associated with leptomeningeal involvement which is best seen on postcontrast MRI [49, 58]. Our data showed that there are high APTw signals in gadolinium-enhancing pia-arachnoid, which can be attributed to the inflammatory infiltration with increased inflammatory cell density. The potential explanations of the APTw signal sources in infectious lesions serve as a working hypothesis. The exact mechanisms still require further study in the future.

The present study reveals that APTw provides comparable results to Gd-T1w MRI in pediatric intracranial infection. However, the spatial resolution and signal-to-noise ratio of APTw were relatively suboptimal compared to SE/FSE imaging, four small enhancing abscesses on Gd-T1w that could not be seen on APTw. Furthermore, a hyperintense APTw finding does not necessarily overlap with contrast enhancement. There are some false positives. Large vessels would typically demonstrate high APTw signals. Fortunately, large vessels are often evident on standard structural MRI sequences (such as T2w and T1w). When reviewing APTw images, referring to routine structural MR images to identify “hyperintensity artifacts,” such as large vessels, is necessary for accurate interpretation.

When an RF saturation power (B_1_) of 2 *μ*T is used, the APTw signal of the normal brain tissue is almost zero, due to the presence of a negative MTR′_asym_(3.5 ppm) [59]. Exactly, according to our previous study [36], mean APTw intensity values were between -0.91 and 1.29% in the frontal white matter, occipital white matter, and centrum semiovale and between 0.35 and 1.42% in the deep subcortical nuclei (head of caudate nucleus, putamen, and thalamus). Thus, using this power and sufficient saturation time, the APTw images will be homogenous for most normal brain areas, allowing detection of hyperintense APTw signals in the Gd-enhancing rim of the abscess (mean APTw intensity values = 2.24%). Using a rainbow color scale, this leads to a green background with yellow/orange/red hyperintensities, convenient for clinical assessment. Like other MRI sequences, artifacts also appear in APTw MRI [20]. The APTw signal is usually measured by the MTR asymmetry analysis between signal intensities of ±3.5 ppm with respect to the water frequency. Consequently, the quality of APTw imaging greatly depends on the B_0_ homogeneity over the volume imaged, which affects the water resonance position. The local motion or large B_0_ deviation may cause artifacts in the APTw images, most of which can be removed inside the brain through the realignment of the water center frequency, but some signal hyper- and hypointensities are sometimes found in ventricles or in liquefactive necrosis, which should not be confused with APTw effects.

This study has showed some preliminary results of APTw effect of intracranial infection in children. There were some limitations to this study. First, the number of individuals was relatively small in this study. A future study with a large number is needed to better assess infectious lesions of different aetiologies. Second, the wide age range in this study would introduce variations in the APTw that may not be solely due to the disease itself but also due to the natural physiological changes in children's brains during development. Thus, the future study with a large number of individuals should further categorize the results into age groups for analysis. Third, we used 2D single-slice APTw sequences in this study. Our experimental results showed that this single-slice acquisition protocol can provide high-quality B_0_ magnetic field inhomogeneity corrected APTw image with a scan time of only about 3 minutes, so MRI scan can be done in a clinically limited scan time and provide sufficient signal-to-noise ratio. In a planned future study, 3D APTw technology [26, 60] would be used to improve the coverage of the lesion, providing more information and better signal-to-noise ratio. Fourth, when the MTR_asym_(3.5 ppm) is used, the APTw imaging signal may be contaminated more or less by the upfield fat artifact, particularly under the echoplanar imaging acquisition [61]. The new quantitative APT-MRI technique that is able to overcome this limitation such as the extrapolated semisolid MT reference (EMR) approach [62] may be used in our future study. Finally, the APTw signal quantified from MTR_asym_(3.5 ppm) is contaminated by the upfield NOE signals (including the conventional magnetization transfer asymmetry) [44, 63]. To quantify a pure APT signal, several alternative APTw imaging acquisition approaches [64, 65] or analysis [66–68] approaches have been proposed. However, these methods require a longer scan time, and their use for the routine clinical practice requires further validation. Furthermore, the APTw metric is affected by several factors, including amide proton concentration, tissue pH, water proton concentration, and T1 of water [69]. However, according to the previous papers [19, 70], the contributions of tissue water content and T1 to APTw signal are mostly compensated in many diseases. Notably, some recent studies [71, 72] have clearly indicated that the APT effect in tissue is actually not affected by water T1 at the saturation power of 2 *μ*T, as used in this study.

## 5. Conclusions

In conclusion, the present study evaluated, for the first time, pediatric intracranial infection with APTw imaging. Our study has shown that the lesion hyperintensity on APTw is significantly related to lesion enhancement on Gd-T1w in children with intracranial infection (including brain abscess, viral encephalitis, and meningitis). These initial data show that APTw MRI is a noninvasive technique for the detection and characterization of an intracranial infectious lesion. APTw MRI is based on endogenous contrast agents, so no contrast agent injection is required. Although APTw may not replace Gd-T1w to demonstrate intracranial infection, it may serve as a screening MRI sequence where the use of GBCAs is a concern. This represents a promising step forward to reduce contrast agent load and the need for intravenous access, which is especially beneficial for pediatric cases.

## Figures and Tables

**Table 1:** Patient demographic data.

Case no.	Age (months)/sex	Diagnosis	The pathogen of infection	Gadolinium enhancement
1	86/F	Tuberculous abscess	Tubercle bacillus	Yes
2	42/F	Tuberculous abscess	Tubercle bacillus	Yes
3	14/M	Pyogenic abscess	Staphylococcus aureus	Yes
4	84/M	Pyogenic abscess	Streptococcus pneumoniae	Yes
5	128/F	Pyogenic abscess	Streptococcus pneumoniae	Yes
6	162/M	Pyogenic abscess	Streptococcus pneumoniae	Yes
7	56/F	Pyogenic abscess	Staphylococcus aureus	Yes
8	1/F	Pyogenic abscess	Escherichia coli	Yes
9	129/M	Pyogenic abscess	Streptococcus intermedius	Yes
10	2/M	Pyogenic abscess	Bacteroides fragilis	Yes
11	9/F	Fungal abscess	Candida albicans	Yes
12	104/M	Fungal abscess	Exophiala	Yes
13	17/M	Viral encephalitis	Coxsackievirus	No
14	75/F	Viral encephalitis	Epstein-Barr virus	No
15	82/F	Viral encephalitis	Coxsackievirus	No
16	58/M	Viral encephalitis	Epidemic encephalitis B virus	No
17	40/M	Viral encephalitis	Epstein-Barr virus	No
18	17/F	Viral meningoencephalitis	Coxsackievirus	Yes
19	15/M	Viral meningoencephalitis	Human herpesvirus 7	Yes
20	16/M	Viral meningoencephalitis	Herpes simplex virus	Yes
21	7/M	Tuberculous meningitis	Tubercle bacillus	Yes
22	163/M	Tuberculous meningitis	Tubercle bacillus	Yes
23	5/M	Tuberculous meningitis	Tubercle bacillus	Yes
24	46/F	Tuberculous meningitis	Tubercle bacillus	Yes
25	11/F	Pyogenic meningitis	Streptococcus pneumoniae	Yes
26	123/F	Pyogenic meningitis	Staphylococcus epidermidis	Yes
27	2/M	Pyogenic meningitis	Escherichia coli	Yes
28	9/F	Pyogenic meningitis	Haemophilus influenzae	Yes

**Table 2:** APTw and MTR values for each tissue type of brain abscess (%; mean value ± SD).

Variable	Gadolinium-enhancing rim	Perifocal edema	CNABT	*P* values^a^
APTw	2.24 ± 0.36	1.10 ± 0.25	0.49 ± 0.12	**<0.001**, **<0.001**, **<0.001**
MTR	15.28 ± 1.70	15.85 ± 2.42	20.38 ± 3.71	0.786, **<0.001**, **<0.001**

Note: CNABT = contralateral normal-appearing brain tissue. ^a^Three post hoc *P* values corresponded to those between gadolinium-enhancing rim and perifocal edema, gadolinium-enhancing rim and CNABT, and perifocal edema and CNABT, respectively. Bold indicates a significant change.

**Table 3:** Comparisons of APTw and MTR values between brain abscess and viral encephalitis (%; mean value ± SD).

Variable	Brain abscess (gadolinium-enhancing rim)	Viral encephalitis	*P* values
APTw	2.24 ± 0.36	0.92 ± 0.35	**<0.001**
MTR	15.28 ± 1.70	25.98 ± 3.22	**<0.001**

Note: bold indicates a significant change.

**Table 4:** Detection of infectious lesions with conventional MR prior to intravenous contrast administration versus APTw.

		APTw	
		+	−
Infectious lesions (*n* = 84)	Gd-T1w		
	+	56	4
	−	2	22
	Sens.	93.3%	
	Spe.	91.7%	
	PPV	96.6%	
	NPV	84.6%	
	*κ* value^a^	0.83	
	*P* value^b^	0.69	

Note: Sens. = sensitivity; Spe. = specificity; PPV = positive predictive value; NPV = negative predictive value. ^a^Excellent agreement, *κ* > 0.75; fair to good agreement, *κ* = 0.40–0.75; poor agreement, *κ* < 0.40. ^b^Calculated with the McNemar test.

## Data Availability

All data used to support the findings of this study are included within the article.

## Abbreviations

ADC: Apparent diffusion coefficient
APT: Amide proton transfer
APTw: Amide proton transfer-weighted
CEST: Chemical exchange saturation transfer
CNABT: Contralateral normal-appearing brain tissue
CSF: Cerebrospinal fluid
DWI: Diffusion-weighted imaging
FLAIR: Fluid-attenuated inversion recovery
GBCA: Gadolinium-based contrast agent
Gd: Gadolinium
Gd-T1w: Gadolinium-enhanced T1-weighted imaging
MT: Magnetization transfer
MTR: Magnetization transfer ratio
MTR_asym_: Magnetization transfer-ratio asymmetry
NPV: Negative predictive value
PPV: Positive predictive value
RF: Radiofrequency
ROI: Region of interest.
